# Video education improves patients’ knowledge and satisfaction in treatment of solar lentigines with picosecond 755-nm alexandrite laser: A retrospective study

**DOI:** 10.3389/fmed.2023.1158842

**Published:** 2023-04-27

**Authors:** Jia-Wei Liu, Yan Tan, Tian Chen, Yue-Tong Qian, Tao Zhang, Dong-Lai Ma

**Affiliations:** Department of Dermatology, Peking Union Medical College Hospital, Chinese Academy of Medical Sciences and Peking Union Medical College, National Clinical Research Center for Dermatological and Immune Diseases, Beijing, China

**Keywords:** video education, informed consent, dermatological laser treatment, patient satisfaction, picosecond laser, elderly population

## Abstract

**Background:**

Picosecond lasers are widely used in dermatologic and cosmetic practice. In clinical practice, informed consent for laser treatments is critical to ensure patients’ understanding of health information.

**Objectives:**

To evaluate whether video-based informed consent improves patient comprehension and satisfaction.

**Methods:**

The study was performed from August 1 to November 30, 2022. Solar lentigines patients who fulfilled the inclusion criteria were included. Before October 1, 2022, traditional informed consent methods were performed. In the subsequent 2 months, a video-based informed consent was used as an adjunct to traditional consenting methods. Finally, patient comprehension of relevant knowledge about laser treatment and client satisfaction were assessed.

**Results:**

A total of 106 patients were included. The mean number of correct answers in the comprehension assessment in the video-based informed consent group was significantly higher than that in the traditional informed consent group (4.4 ± 1.2 vs. 3.4 ± 1.1, *p* < 0.001). Compared to the traditional informed consent group, more correct answers in the video-based informed consent group were provided by older patients (3.9 ± 1.2 vs. 2.9 ± 1.1, *p* = 0.004) and patients with lower education levels (4.1 ± 1.1 vs. 3.0 ± 1.2, *p* < 0.001). The mean satisfaction score in the video-based informed consent group was significantly higher than that in the traditional informed consent (27.8 ± 5.7 vs. 24.3 ± 6.2, *p* = 0.003).

**Conclusion:**

Video-based informed consent helps patients learn clinical literacy more effectively and improves patient satisfaction, especially in those with lower education levels and older ages.

## 1. Introduction

Solar lentigo is characterized by a pigment change, such as dark brown spots in the skin, which occurs due to exposure to ultraviolet (UV) radiation (1). UV radiation can lead to locally proliferating melanocytes and the accumulation of melanin in skin cells (keratinocytes) (1). Recently, laser treatment targeting of the melanin pigment has been frequently used to treat solar lentigo in cosmetic dermatology (2). Removing solar lentigines using picosecond lasers is an effective, accessible, and fast method, which generally requires one or two treatments. Currently, picosecond lasers are mainly available in dermatology to treat a wide range of cutaneous conditions with good efficacy, although this technique was originally designed for tattoo removal (1). In addition to using picosecond in solar lentigo, many lasers are increasingly being applied in skin disease, such as fractional ablative and nanosecond lasers (3, 4). It is reported that skin treatment (Intense Pulsed Light, Combination Lasers) has increased to 280,815 in the United States alone (5).

Taking the use of picosecond lasers in treating solar lentigo as an example, it is generally safe and can selectively remove the excess pigment and produce a reborn layer of non-pigmented skin cells in response to healing without influencing the surrounding skin tissue (1). In addition, picosecond lasers have the advantage of producing a photomechanical effect (photoacoustic) with higher compartmentalized peak temperature generation (6), but several common side effects, such as pain, crusting, erythema, scarring, blistering, and post-inflammatory hyperpigmentation, have been reported that are more severe with higher treatment power (7).

Informed consent for laser treatments is a critical component of clinical practice. There are specific legal requirements for the accompanying documentation and what must be disclosed to patients. However, whether the process is smooth for patients depends on their understanding of health literacy. Patients’ comprehension of medical conditions and available interventions is valuable since it incorporates an individual’s abilities to acquire and integrate information (8). In addition, a thorough understanding of health literacy is a premise for effective communication between patients and dermatologists, which improves their medication compliance, health outcomes, and satisfaction (9–11). Therefore, this study intended to investigate: (1) the level of comprehension of solar lentigo patients regarding basic literacy in lasers treatments, including expectations and post-treatment instructions; (2) whether video-based informed consent could improve patient comprehension and satisfaction in the era of information and network; and (3) provide clinical data and evidence for dispensing medical information in a new way, such as videos, cartoons, or smartphone applications to improve clinical outcomes.

## 2. Materials and methods

This retrospective study was performed from August 1, 2022 to November 30, 2022. The approval was obtained from the Ethics Committee of the author’s affiliation (Ethical No: S-K1687) following the Declaration of Helsinki. Patients were informed about the aims of the study and provided written consent.

### 2.1. Study population

All patients presented to our outpatient cosmetic clinic who are more than 18 years old are eligible for study participation.

Inclusion criteria were as follows: (1) patients with clinically diagnosed and dermatoscopically confirmed solar lentigines; (2) chose picosecond 755-nm alexandrite laser treatment; and (3) the ability to completely understand the instructions and answer the questionnaires.

The excluded criteria were: (1) unwillingness to participate the study; (2) contraindications of laser therapy, such as photosensitive diseases and autoimmune diseases; (3) being unable to use the video-based informed consent due to illness such as blindness; and (4) the patient who previously received picosecond laser treatment.

A total of 153 patients were invited to participate in the study. 37 patients declined to participate after being fully explained about the aim and procedure of the study. Additionally, 10 patients were ruled out as they did not meet the inclusion and excluded criteria.

### 2.2. Procedure

During the routine practice, each individual underwent a detailed medical history enquiry and physical examination performed by a senior dermatologist to determine the diagnosis. Available treatment options and informed consent concerning potential risks from laser treatments were also elaborated. Topical anesthetic agents (Compound lidocaine cream, 2.5% lidocaine, and 2.5% propitocaine, Tongfang Pharmaceutical Group Co., Ltd., Beijing) were used on the surface of targeted skin areas under cling-film occlusion for 30–60 min prior to laser treatment. All the enrolled patients were treated with the same picosecond 755-nm alexandrite laser device (Picosure; Cynosure, MA). Treatment spot size, frequency, fluences, and pulse duration were determined based on the dermatologist’s clinical discretions. Patients were recommended to use sunscreen (SPF ≥ 30) and avoid exposure to sunlight. The clinical endpoint depended on the immediate whitening of the targeted pigmented lesions. All the patients underwent treatments at 4–6 weeks intervals, according to the specific clinical response.

Before October 1, 2022, informed consent regarding laser treatments was performed traditionally, using verbal and written methods to convey health information. In the subsequent 2 months, a video developed *via* cartoon-based format to visualize and simplify health information to perform the informed consent for laser treatments and relevant post-treatment instruction was used as an adjunct to traditional consenting methods. The video was created with Harmony 22 software (Toom Boom Animation Inc., MTL, CAN) by a professional animation company (Zhongzhiliancheng, BJ, CHN). A visual snippet is included in the appendix (Supplementary material). This video included information, such as: suitable diseases for picosecond laser treatment, what to do before the laser treatments (e.g., cost, psychological preparation); associated benefits, expectations, and risks; and post-treatment care and instruction. In the traditional group, the informed consent was completed verbally, physically, and in writing, such as through a packet of notifications and sheets. In the video-based informed consent group, dermatologists briefly went through the traditional informed consent process with the patients before providing them with a QR code linked to the education video. Patients were then able to scan the QR code with their smartphone and watch the video. All participants were asked to complete a Patient Comprehension Assessment on relevant knowledge about laser treatment, as well as a Client Satisfaction Questionnaire.

### 2.3. Instruments

A dermatologist responsible for reviewing the instruments was blinded to the patient group.

#### 2.3.1. Patient comprehension assessment

The assessment included demographic and comprehension of health information about laser treatments. The demographic section contained four questions (gender, age, education level, and socioeconomic status). Meanwhile, the patient comprehension section included six questions to examine the understanding of relevant health information (Table 1). The comprehension questions were structured as four-option multiple choices.

**Table 1:** Patient comprehension assessment.

Questions	Options
Which of the following skin disease can be treated by picosecond laser?	Skin dark spots Eczema Acne Vitiligo
When can I get my treatment area wet?	3 days after treatment 1 week after treatment Immediately after treatment I do not know
How long should I strictly protect myself from the sun after treatment?	For 1 month A week or two 3 months or longer I do not know
What kind of sun protection can I use during this time period?	Use hats or umbrellas Wear Sunscreen > 30 SPF Avoid direct sun exposure activities such as out-door running or swimming All the above
When will the scales fall off?	7–10 days 3 days 1 months I do not know
Which of the following activity benefits the skin recovery after laser therapy?	Ice packing after treatment Have a Sauna Swimming All the above

#### 2.3.2. Client satisfaction questionnaire-8

The Client Satisfaction Questionnaire (CSQ-8) was completed at the end of treatment and comprised eight patient-reported questions assessed on a four-point Likert scale (12). A maximum score of 32 means the highest satisfaction, while the minimum score is 8, representing the poorest satisfaction. The CSQ-8 has been widely used to examine patient satisfaction with consenting methods in previous studies with good reliability and validity (13).

### 2.4. Statistical analysis

Continuous data were expressed as mean ± standard deviation (SD) and compared using the *t*-test. Categorical data were summarized as a percentage and compared with the χ^2^-test. A two-tailed *p* < 0.05 was considered a statistically significant difference. The cut-off values for the continuous variables were determined using mean values. Logistic regression was conducted to estimate the odds ratio (OR) and 95% confidence interval (CI) to determine the risk factors for poor comprehension scores in patients with solar lentigo. Then, multivariable logistic models were used to eliminate the variables from univariate analysis through a stepwise approach. All statistical analyses were performed using SAS 9.1 (SAS Institute, Cary, NC, United States).

## 3. Results

A total of 106 patients responded to the study, with an average age of 58.5 years (SD = 9.5). About 72.6% of patients (77/106) were female, and 52.8% (56/106) constituted the group of patients who received video-based informed consent adjunct to the traditional approach concerning laser treatments. The rest of the patients were in the group that received informed consent through the traditional approach. The basic characteristics of the enrolled patients are shown in Table 2.

**Table 2:** Demographics of study patients.

	Patients with video-based informed consent	Patients with traditional informed consent	*p* value
Number of patients	56	50	
Gender (Female), N (%)	41(73.2%)	35(70.0%)	0.872
Age (years), mean ± stand deviation	57.7 ± 9.2	59.4 ± 9.6	0.354
Education level < bachelor degree, N(%)	36(64.3%)	31(62.0%)	0.967
*Annual household income, N(%)*			
<$ 22,000	27(48.2%)	26(52.0%)	0.846
≥$22,000	29(51.8%)	24(48.0%)	

### 3.1. Analysis for comprehension assessment

The video-based informed consent group answered more questions correctly than the traditional informed consent group in the comprehension assessment, regardless of age, gender, education level, and annual income (all *p* < 0.05, Table 3). Compared to the mean number of correct answers in the comprehension assessment in the video-based informed consent group, that in the traditional informed consent group was significantly lower (4.4 ± 1.2 vs 3.4 ± 1.1, *p* < 0.001). In addition, the percentage of patients who answered all the questions correctly *via* traditional informed consent was significantly lower than that *via* video-based informed consent (48.2% [27/56] vs. 28.0% [14/50], *p* = 0.033). Compared to video-based informed consent, traditional informed consent failed to assist more patients in answering each question correctly (59.5% [200/336] vs. 49.3% [148/300], *p* = 0.020).

**Table 3:** Comparisons of mean correct number of comprehension assessment in video-based informed consent across variables.

	Gender			Age			Education level < bachelor degree			Annual income			Female	Male	*p* value	≥65 years	<65 years	p value	≥Bachelor degree	<Bachelor degree	*p* value	<$22,000	≥$22,000	*p* value
Number (%)	41(73.2%)	15(26.8%)		26(46.4%)	30(53.6%)		20(35.7%)	36(64.3%)		27 (48.2%)	29(51.8%)	
Mean correct number of comprehension assessment in video-based informed consent	4.3 ± 1.0	4.5 ± 1.3	0.544	3.9 ± 1.2	4.9 ± 1.1	0.002^*^	5.0 ± 0.8	4.1 ± 1.1	0.002^*^	4.0 ± 0.9	4.8 ± 1.2	0.007^*^
Number (%)	35(70.0%)	15(30.0%)		24(48.0%)	26(52.0%)		19(38.0%)	31(62.0%)		26(52.0%)	24(48.0%)	
Mean correct number of comprehension assessment in traditional informed consent	3.5 ± 1.0	3.3 ± 1.3	0.557	2.9 ± 1.1	3.8 ± 1.3	0.011^*^	3.9 ± 1.1	3.0 ± 1.2	0.011^*^	3.1 ± 1.0	3.7 ± 1.1	0.049^*^
*p* value	0.001^*^	0.017^*^		0.004^*^	0.001^*^		0.001^*^	<0.001^*^		0.001^*^	0.001^*^	

Mean ± standard deviation was shown. *means statistically significant.

In both groups, more correct answers were provided by younger patients (video-based informed consent: 3.9 ± 1.2 vs. 4.9 ± 1.1, *p* = 0.002; traditional informed consent: 2.9 ± 1.1 vs. 3.8 ± 1.3, *p* = 0.011), those with higher education levels (video-based informed consent: 4.1 ± 1.1 vs. 5.0 ± 0.8, *p* = 0.002; traditional informed consent:3.0 ± 1.2 vs. 3.9 ± 1.1, *p* = 0.011), and those with a higher annual income (video-based informed consent: 4.0 ± 0.9 vs. 4.8 ± 1.2, *p* = 0.007; traditional informed consent: 3.1 ± 1.0 vs. 3.7 ± 1.1, *p* = 0.049) compared to their counterparts (Table 3). However, there were no significant differences in gender (video-based informed consent: 4.3 ± 1.0 vs. 4.5 ± 1.3, *p* = 0.533; traditional informed consent: 3.5 ± 1.0 vs. 3.3 ± 1.3, *p* = 0.554). More correct answers in the video-based informed consent group were provided by older patients (3.9 ± 1.2 vs. 2.9 ± 1.1, *p* = 0.004) and patients with lower education levels (4.1 ± 1.1 vs. 3.0 ± 1.2, *p* < 0.001), compared to their counterparts in traditional informed consent group (Table 3).

### 3.2. Analysis of patient satisfaction

The mean CSQ-8 score in the video-based informed consent group was significantly higher than that in the traditional informed consent (27.8 ± 5.7 vs. 24.3 ± 6.2, *p* = 0.003). Patients who watched the video about informed consent more than three times reported higher CSQ-8 scores than those who watched it less than three times (29.3 ± 6.0 vs. 23.5 ± 6.5, *p* = 0.002). All patients in the video-based informed consent group reported that their health literacy would improve using such an approach.

### 3.3. Logistic regression analysis of poor satisfaction with the laser treatments

Univariate logistic regression analysis showed that the traditional informed consent group (*OR* = 2.6, 95% *CI* = 2.0–3.0, *p* = 0.012), age ≥ 35.0 years (*OR* = 1.8, 95% *CI* = 1.1–2.5, *p* = 0.023), level of education below university (*OR* = 1.7, 95% *CI* = 1.2–2.2, *p* = 0.013), and annual income below $22,000 (*OR* = 1.4, 95% *CI* = 1.0–1.8, *p* = 0.034) were predictors of poor satisfaction with the laser treatments.

After adjusting for age, sex, level of education less than a bachelor’s degree (adjusted *OR* = 2.1, 95% *CI* = 1.6–2.6, *p* = 0.025), and annual income below $22,000 (adjusted *OR* = 1.8, 95% *CI* = 1.4–2.2, *p* = 0.029), traditional informed consent (adjusted *OR* = 2.9, 95% *CI* = 2.4–3.4, *p* = 0.037) remained a predictor for poor satisfaction in the multivariate logistic regression analysis.

## 4. Discussion

The level of understanding of health information is indicative of patients’ knowledge of their medical conditions. The results showed that video interpretation for health literacy regarding laser treatment improved the ability to grasp crucial health information, such as pre-treatment preparation, complications, and after-treatment routine care. Compared to the mean number of correct answers in the comprehension assessment in the video-based informed consent group, that in the traditional informed consent group was significantly lower (4.4 ± 1.2 vs. 3.4 ± 1.1, *p* < 0.001). In addition, the percentage of patients who answered all the questions correctly *via* traditional informed consent was significantly lower than that *via* video-based informed consent (48.2% [27/56] vs. 28.0% [14/50], *p* = 0.033). Moreover, health literacy is crucial to ensure satisfactory treatment outcomes. Patient satisfaction, reflected by the CSQ-8 score, in the video-based informed consent group was significantly higher than that in the traditional informed consent group (27.8 ± 5.7 vs. 24.3 ± 6.2, *p* = 0.003).

Even after controlling for age, sex, education level, and income, patients who use video-based informed consent showed higher satisfaction with laser treatment than those with traditional informed consent. Older patients and patients with lower levels of education had fewer barriers to understanding their clinical encounters with video-based informed consent. The widespread use of smartphones has opened up new avenues for patient education in the information age. Recently, video- or multimedia-assisted informed consent has been applied in several clinical practices, such as cataract surgery, central venous catheter procedure, trauma surgery, and endoscopy (14–17). Sowan et al. (14) found high satisfaction with both a video and a traditional informed consent. In addition, Tipotsch-Maca et al. (15) reported that videos can improve patient satisfaction. Another study showed that a video about adequate informed consent for surgery, developed using a scientific method that integrated the opinions of different stakeholders, particularly patients, was a useful tool in improving the knowledge and satisfaction of trauma patients in the emergency department. However, such a promising tool has never been discussed in dermatology (17). Another notable advantage was that patients who completed their consent using videos reported higher satisfaction. We assumed that videos could allow patients to view the information repeatedly until they thoroughly understand their clinical encounter. A thorough understanding of the clinical encounter helps reduce nervous and unsecured feelings, thus improving patient satisfaction. This assumption is supported by the fact that patients who watched the video about informed consent over three times reported higher CSQ-8 scores than those who watched it less than three times. Besides, videos in the form of cartoons and pictures can show detailed and vivid descriptions of the benefits and potential risks of laser treatments, which improves understanding of medical terminology. For example, the possible complications which may occur after laser treatment and need to be treated in the hospital, like “blisters,” “pigmentation,” and “scars.”

In addition, we found that video-based informed consent was less time-consuming for medical staff. When we used video-based informed consent, dermatologists or nurses only required an average of 1–2 min to give brief instructions before patients got ready to watch the video. The verbal pulse video approach made the process of patient consent more efficient. The novel COVID-19 pandemic undoubtedly created enormous stress throughout the healthcare system, and dermatology is no exception. Preventive measures and precautions have been put into immediate practice, including an international travel limit, social distancing, online education systems, and a campaign that encourages everyone to “stay-at-home.” Due to of the “shelter-in-place” guidelines, the number of patient requests for dermatology outpatient clinic visits has sharply reduced (18), which will eventually lead to the accumulation of patients with dermatologic diseases after the pandemic. In response to this unusual situation, we believe telemedicine, also termed more broadly telehealth, can help offer uninterrupted clinical instruction and supportive care to patients who have access to a smartphone or the Internet. In such a background, the transition from conventional face-to-face communication between patients and doctors toward telecommunication of treatment plans and informed consent is being accelerated. Informed consent is an integral part of telemedicine. The present findings also provide preliminary evidence for the benefits of video-based informed consent. Adapting video-based informed consent is promising in improving satisfaction and understanding health information in the telemedicine era.

Video-based informed consent has been shown to have good acceptance rates among patients, but it is not without its drawbacks. During our study, we encountered a case where a patient reported experiencing unpleasantness, after viewing the content about blister formation. This adverse impact resulted in the patient refusing to undergo the treatment procedure. This case has raised concerns that the video display format may inadvertently exaggerate the side effects, potentially causing unnecessary anxiety and apprehension in patients. Therefore, it is imperative that dermatologists carefully design video content with the patient’s perspective in mind to avoid triggering unnecessary nervousness and apprehensiveness. In addition, costs associated with creating high-quality video content is another concern. The production of animated videos for medical procedures can be expensive, costing up to $500 per minute. The cost issue can hinder medical institutions and healthcare providers from using this method to explain complex medical procedures.

In conclusion, video-based informed consent help patients have access to clinical instruction more effectively. Specifically, delivering health literacy by video contributed to a better patient comprehension of the presented health information. More importantly, patients with lower education levels and older patients who generally have poorer health literacy can also achieve better understanding. Although laser treatments have shown to be safe among the general population (7, 19), old age and low education level, especially with illiterate patients, did have a direct negative effect on patient satisfaction, comprehension, and recall of the relevant health information (20). Adapting video-based informed consent in the clinical practice shows the potential to improve patient satisfaction and reduce the risk of adverse events. By providing patients with a clear and concise understanding of the procedure and associated risks, healthcare providers can facilitate better communication, leading to improved patient outcomes. Moreover, during this challenging COVID-19 pandemic, video-based informed consent as an effective alternative helps in minimizing dissemination of the virus and avoiding more collateral damage due to “stay at home” orders. Meanwhile, this instrument can decrease nurses’ and doctors’ working time for patient education, instruction, and informed consent, reducing their workload. However, more efforts are needed to optimize the video’s informative value and efficacy with improving patients’ abilities to remember and understand health information concerning laser treatments and associated risks.

This study had several limitations that should be noted. First, the present survey unilaterally investigated the effect of video-based informed consent on patient understanding. However, its influence on nurses and dermatologists was not examined in the present study, although we did experience reduced workload and work time in practice. Second, the items in our comprehension assessment only contained 10 questions, which may not completely capture patients’ level of understanding of their clinical encounters. Third, the “multiple choice” questions used in the present study design, instead of “true or false,” are more effective in examining responders’ comprehension. However, the correct answer for the “multiple choice” may be due to blind guessing.

## Data availability statement

The raw data supporting the conclusions of this article will be made available by the authors, without undue reservation.

## Ethics statement

The studies involving human participants were reviewed and approved by the Ethics Committee of the Peking Union Medical College Hospital. The patients/participants provided their written informed consent to participate in this study.

## Author contributions

D-LM designed the study. J-WL, TC, Y-TQ, TZ, and YT perform the data collection and analysis. J-WL drafted the original manuscript, and J-WL, YT, TC, YTQ, and D-LM participated in in reviewing and revising. D-LM supervised the whole study. All authors contributed to the article and approved the submitted version.

## Conflict of interest

The authors declare that the research was conducted in the absence of any commercial or financial relationships that could be construed as a potential conflict of interest.

## Publisher’s note

All claims expressed in this article are solely those of the authors and do not necessarily represent those of their affiliated organizations, or those of the publisher, the editors and the reviewers. Any product that may be evaluated in this article, or claim that may be made by its manufacturer, is not guaranteed or endorsed by the publisher.
